# Draft Genome Sequence of a “*Candidatus* Brocadia” Bacterium Enriched from Activated Sludge Collected in a Tropical Climate

**DOI:** 10.1128/genomeA.00406-18

**Published:** 2018-05-10

**Authors:** Xianghui Liu, Krithika Arumugam, Gayathri Natarajan, Thomas W. Seviour, Daniela I. Drautz-Moses, Stefan Wuertz, Yingyu Law, Rohan B. H. Williams

**Affiliations:** a Singapore Centre for Environmental Life Sciences Engineering, Nanyang Technological University, Singapore; b School of Civil and Environmental Engineering, Nanyang Technological University, Singapore; c Singapore Centre for Environmental Life Sciences Engineering, National University of Singapore, Singapore

## Abstract

Here, we present the draft genome sequence of an anaerobic ammonium-oxidizing bacterium (AnAOB), “*Candidatus* Brocadia,” which was enriched in an anammox reactor. A 3.2-Mb genome sequence comprising 168 contigs was assembled, in which 2,765 protein-coding genes, 47 tRNAs, and one each of 5S, 16S, and 23S rRNAs were annotated. No evidence for the presence of a nitric oxide-forming nitrite reductase was found.

## GENOME ANNOUNCEMENT

Anaerobic ammonium oxidation (anammox) is a microbial bioprocess in which ammonium (NH_4_^+^) and nitrite (NO_2_^−^) are converted to dinitrogen (N_2_) gas via the generation of hydrazine (N_2_H_4_) (1), and it is considered a more energy efficient and effective N removal bioprocess than conventional wastewater treatment systems (1). All known anaerobic ammonia-oxidizing bacteria (AnAOB) belong to the phylum Planctomycetes, with five “*Candidatus*” genera defined (1, 2). We established an enrichment protocol to target anammox organisms in a sequencing batch reactor seeded with return activated sludge from a water reclamation plant (Public Utilities Board, Singapore). The reactor was fed synthetic wastewater containing ammonium and nitrite and operated at 35°C. Within 6 weeks, simultaneous ammonium and nitrite depletion was observed, along with the appearance of a reddish-brown biofilm on the walls of the reactor, which is highly characteristic of anammox biofilms (1). Fluorescent *in situ* hybridization (FISH) analysis conducted on day 120 postinoculation, using the Amx820 probe (3), indicated the presence of anammox bacteria. The wall biofilm was then sampled, and shotgun metagenomics was performed on an Illumina MiSeq run using 300-bp paired end sequencing (Illumina). Following read filtering, we analyzed these data using RiboTagger (4) and observed ∼70% relative abundance for genus “*Candidatus* Brocadia” (V4 ribotag annotated against SILVA release 119) (5).

We constructed a metagenome assembly using Newbler v 2.9 (454 Life Sciences) and employed the CONCOCT program to cluster these contigs into 27 putative genome bins (6). The most abundant bin contained 168 contigs (length, 1,527 to 114,900 bp; mean, 19,210 bp) and had a total sequence length of 3.27 Mb, with an average GC content of 42.3%. Analysis with CheckM (7) showed this genome to have a high completeness (98.8%) and low contamination rate (1.7%), and it was annotated to the marker set lineage UID2565, which is selective for “*Candidatus* Brocadiaceae.” We annotated predicted genes with Prokka (v1.10) (8), finding that this draft genome sequence encodes 2,765 genes, 47 tRNAs, and 3 rRNA genes. Using the CheckM SSUfinder module (7), one 16S gene of length 1,572 bp was identified from this bin, which, when analyzed using the SILVA Incremental Aligner (SINA) (9), was annotated to “*Candidatus* Brocadia,” with 94% sequence identity. Collectively, these data suggest that we have recovered a new member of genus “*Candidatus* Brocadia.”

This draft genome sequence encodes gene clusters relating to core anammox metabolism, including 9 multiheme *hdh*/*hao-*like proteins, the three subunits of *hzs* (hydrazine synthase), and a single three-subunit nitrate reductase/oxidoreductase (*nxr*) (10). The presence in anammox bacteria of nitrite reductases, either nitric oxide (NO)-forming and/or ammonia-forming, remains subject to considerable uncertainty (10). Using a sequence similarity network approach that incorporates all protein sequences from (i) the present metagenome assembly, (ii) all extant draft genomes, and (iii) all known nitrate reductases, we identified a gene in the current draft genome that has an 88% average amino acid identity (AAI) and a 100% alignment overlap with ammonia-forming nitrite reductase in the genome of “*Candidatus* Brocadia sinica JPN1” (*E *=* *1 × 10^−257^) and, at a lower level of support, with an ammonia-forming nitrite reductase in genus Rhodopirellula (53% AAI; 94% alignment overlap; *E *=* *1 × 10^−146^). We did not find any clear evidence for the presence of an NO-forming *nir* gene, consistent with results of previous investigations of the genus “*Candidatus* Brocadia” (10–13).

### Accession number(s).

The draft genome described in this paper has been deposited at DDBJ/ENA/GenBank under the accession number PJZV00000000. The raw sequence data from the reactor community are available via NCBI BioProject accession number PRJNA383754.
